# A multiplex PCR for differential detection of *Echinococcus granulosus* sensu stricto, *Echinococcus multilocularis* and *Echinococcus canadensis* in China

**DOI:** 10.1186/s40249-019-0580-2

**Published:** 2019-07-30

**Authors:** Jing-Ye Shang, Guang-Jia Zhang, Sha Liao, Yan Huang, Wen-Jie Yu, Wei He, Guang-You Yang, Tiao-Ying Li, Xing-Wang Chen, Bo Zhong, Qian Wang, Qi Wang, Rui-Rui Li, Hao Wang

**Affiliations:** 1 Institute of Parasitic Diseases, Sichuan Center for Disease Control and Prevention, Chengdu, Sichuan People’s Republic of China; 2 Department of Parasitology, College of Veterinary Medicine, Sichuan Agricultural University, Chengdu, Sichuan People’s Republic of China

**Keywords:** Echinococcosis, *Echinococcus granulosus* s. s., *Echinococcus multilocularis*, *Echinococcus canadensis*, Multiplex PCR

## Abstract

**Background:**

Echinococcosis caused by *Echinococcus* is one of the most major infectious diseases in north-west highland of China. *E. granulosus* sensu strict, *E. multilocularis*, and *E. canadensis* are known to be the only three species related to human health transmitting in the areas. To achieve targeted treatment and control of echinococcosis, the accurate identification and discrimination of the species are important. However, currently the available diagnostic approaches do not present ideal results either in accuracy or efficiency.

**Methods:**

In the study, a set of primers were designed to aim at the three human-pathogenic *Echinococcus* species in China. The one-step multiplex PCR assay was developed and evaluated for the specificity and sensitivity. A total of 73 parasitic lesions and 41 fecal materials obtained from human and various animals collected in the clinic and the field were tested to assess the applicability of this method.

**Results:**

The multiplex PCR effectively detected the individual DNA from the targeted species and their random mixtures generating with distinguishable expected size of products. The detection limit of the assay for each of the three species was 5 pg/μl when they were tested separately. When DNA mixtures of the targeted species containing the same concentration were used as templates, the lowest amount of DNA which can be detected was 50 pg/μl, 10 pg/μl and 5 pg/μl for *E. granulosus* s. s., *E. multilocularis*, and *E. canadensis* respectively. No cross-reactivity was observed when DNA from eight genetically close species was used as control templates. The multiplex PCR identifications of all samples were in line with the original sequencing results except for those infected with *E. shiquicus*, which showed negative signals in the developed assay. Of all the tested stool materials, 16 were previously found positive for *Echinococcus* by visual and microscopic examination. Among these 16 samples, 13 were confirmed by the multiplex PCR, and the other three tested negative. Additionally, the multiplex PCR identified another 14 positive feces from the remained 25 stool samples which absence of worms.

**Conclusions:**

The developed multiplex PCR shows advantages in fast diagnosis and large-scale epidemiological investigation, which proven to be a promising tool utilized in clinic and surveillance system.

**Electronic supplementary material:**

The online version of this article (10.1186/s40249-019-0580-2) contains supplementary material, which is available to authorized users.

## Multilingual abstracts

Please see Additional file 1 for translations of the abstract into the five official working languages of the United Nations.

## Background

Echinococcosis, caused by the infection of one or more of nine species within the genus *Echinococcus*, is among the most neglected zoonoses distributed globally, bringing about great public health concerns and economic losses [1–3]. In China, where 368 endemic counties distributed in nine provinces with an estimated 166 098 cases nationally [4], four species coexisted [5, 6]. *E. granulosus* sensu strict (G1–3) and *E. canadensis* (G6–8, 10) infections lead to cystic echinococcosis (CE), which is the most major type of the disease [7]. *E. multilocularis* infection result in alveolar echinococcosis (AE), also called as “parasite cancer”, is one of the most lethal parasitic infections in humans [8]. *E. shiquicus*, the sister species of *E. multilocularis* [9], has shown no correlations with human or domestic animal infections so far [10–12]. Because the treatment principles and chemotherapeutic effects vary much between CE and AE [13, 14], and the life cycles, transmission patterns and related risk factors of these species are quite different [15], the accurate detection and identification of the species are extraordinarily vital in effective treatment and targeted control of ecinococcosis.

Imaging examination, immunological detection, histopathological analysis, visual and microscopic observation are the main methods extensively used in clinic and surveillance networks. Unfortunately, the correctness and accuracy of the results determined by the approaches have been shown to be insufficient sometimes [16–19]. Furthermore, none of the diagnostic methods can precisely discriminate the species within *E. granulosus* sensu lato. Incorrect or inaccurate diagnosis can lead to serious and even fatal consequences for patients. Without detailed species-level information, it is hard to evaluate the risk level of the pathogen species transmitted in the environment, so as to adjust the prevention and control strategy in time. PCR-based molecular detections therefore have been adopted to conduct the differential diagnosis of echinococcosis.

Currently, most of the known PCR-related assays, designed to detect single species with singular amplification, or use a generic PCR followed with DNA sequencing/ enzyme reaction for species discrimination, or achieve higher sensitivity with a nested-PCR consisted of two steps, cannot meet the needs of rapid diagnosis or large-scale investigation, especially for areas where multiple *Echinococcus* species are co-distributed. Multiplex PCR, a one-tube amplification assay for simultaneous detection of all targeted species, is fast, labor-saving and cost- effective. It is highly applicable to differentiate the pathogens of echinococcosis in the co-transmitted regions. Some multiplex PCR assays have been developed for the detection of species within *Echinococcus* [20–22]. But none of them were aimed at the simultaneously identification of *E. granulosus* s. s., *E. multilocularis*, and *E. canadensis*.

In this study, we describe the development and evaluation of a multiplex PCR assay for the differential detection of three human-pathogenic *Echinococcus* species having been confirmed present in China.

## Methods

### Sample collection

For the initial primers screening and the validation of sensitivity and specificity, a panel of isolates from parasite lesions and adult worms were used, including *E. granulosus* s. s., *E. multilocularis*, *E. canadensis* (G6/7), *E. canadensis* (G8/10) which are the detection targets, *E. shiquicus*, *Taenia taeniaeformis*, *Hymenolepis diminuta*, *T. pisiformis*, *T. saginata*, *T. solium*, *T. asiatica*, and *T. serialis* used as negative controls (Table 1).

**Table 1:** Cestodes used to establish the multiplex PCR and to evaluate the sensitivity and specificity of this assay

Parasite species	Parasite stage	Host origin
*Echinococcus granulosus* s. s.	Metacestode lesion	Human
*E. multilocularis*	Metacestode lesion	Human
*E. canadensis* (G6/7)	Metacestode lesion	Human
*E. canadensis* (G8/10)	Metacestode lesion	Sheep
*E. shiquicus*	Metacestode lesion	Plateau pika
*Taenia taeniaeformis*	Metacestode lesion	Mouse
*Hymenolepis diminuta*	Adult worm	Mouse
*T. pisiformis*	Metacestode lesion	Rabbit
*T. saginata*	Adult worm	Human
*T. solium*	Adult worm	Human
*T. asiatica*	Adult worm	Human
*T. serialis*	Adult worm	Dog

For the verification of the applicability for the detection of human and animal infections, 36, 14, and 23 parasite lesions derived respectively from echinococcosis patients, infected yaks and small mammals were used, along with 41 stool materials obtained after arecoline purgation of domestic dogs (Table 2).

**Table 2:** Samples used for the assessment of the applicability of the multiplex PCR for the detection of human and animal infections

Host origin	Parasite stage	No. of samples
Human	Metacestode lesion	36
Yak	Metacestode lesion	14
Small mammal	Metacestode lesion	23
Dog	Purged feces	41
Total		77

All the tissue samples were stored in 95% ethanol before use. Stool samples were pre-examined by naked-eye and optical microscope.

### DNA extraction and pre-sequencing

Genomic DNA from tissue and fecal samples were extracted respectively using the DNeasy Blood & Tissue Kit (Qiagen, Germany) and the QIAamp DNA Stool Mini Kit (Qiagen, Germany) according to the manufacturer’s protocols. DNA concentration were determined using a Nano Drop spectrophotometer (Thermo Scientific, USA).

All the parasite samples employed in the study were pre-determined to species-level based on an 880-bp sequence of partial cytochrome c oxidase subunit I (*cox1*) gene [23].

### Primer design and selection

The complete mitochondrial genome sequence of *E. granulosus* s. s. (GenBank accession No. NC_008075.1, KJ559023.1), *E. multilocularis* (No. NC_000928.2, AB018440.2), and *E. canadensis* (G6/7, No. NC_011121.1, AB235847.1, and G8/10, No. AB235848.1, AB745463.1) were retrieved from the National Center for Biotechnology Information database (NCBI). The sequences were aligned using MEGA7 (www.megasoftware.net) [24]. Specific-conserved regions were found and used to design the primer pairs by Oligo 7 (DBA Oligo, Inc., USA) [25]. After checking with NCBI primer-BLAST, the primer candidates which showed no cross-hybridization to non-target fragments were preliminarily tested for specificity by singlex amplification. Primers that yielded only specific products with appropriate length size were combined for multiplex reactions. The sets of primers were evaluated on specificity, sensitivity and capability of simultaneous detection of target species. The ones with desired specificity and sensitivity producing distinguishable size of PCR products were selected and further assessed on applicability using appropriate numbers of clinical and field samples.

### Multiplex PCR

Multiplex PCR was performed in a 25 μl reaction mixture containing 1 × GoTaq Hot Start Green Master Mix (Promega, USA), 6.5 μl of ddH_2_O and 1 μl of DNA template. The final concentrations of the primers used to detect each species are listed in Table 3.

**Table 3:** Primers designed for detection of *Echinococcus granulosus* s. s., *E. multilocularis* and *E. canadensis* in the study

Targeted species	Primer name	Sequences (5′ → 3′)	Expected product length	Final concentration (nmol/L)
*E. granulosus* s. s.	g/f	GTCTGTGTTTCTTACCATTG	811	200
	g/r	GACCCGTACAAACATATATCAAC		
*E. multilocularis*	m/f	TTGTTCTTTGTGTTACTGTAGG	457	600
	m/r	CTATACAGACATTGATTACCATAA		
*E. canadensis*	c/f	GTAAGTCTAAGTTGGTTATTATTCAC	315	200
	c/r	CTTATTAAACAACACAAAAATACTAAATG		

PCR was carried out by an initial denaturation at 94 °C for 2 min, followed by 40 cycles of denaturation at 94 °C for 30 s, annealing at 55 °C for 45 s, extension at 72 °C for 60 s, and a final extension at 72 °C for 5 min in a PCR thermocycler (ABI2720, Applied Biosystems, USA).

Amplified products were checked for size using 2% agarose gel electrophoresis and visualized on a UV transilluminator (Universal Hood II, Bio-Rad, Germany). Positive-PCR products were sent for sequencing to confirm the identity on both strands at Tsingke Biological Technology, China.

### Detection capability of single and mixed infection

Genomic DNA from *E. granulosus* s. s., *E. multilocularis*, *E. canadensis*, and their random mixtures were used in the multiplex PCR to assess the feasibility of this method in detecting and discriminating the targeted species individually and simultaneously.

### Evaluation of specificity

To test the specificity of the reaction, multiplex PCR was performed using DNA templates prepared from the three targeted species, along with another eight different non-targeted parasite isolates belonging to the tapeworm lineage as the same as *Echinococcus*.

### Assessment of sensitivity

Serial dilutions of genomic DNA from *E. granulosus* s. s., *E. multilocularis*, *E. canadensis* (G6/7) respectively, and their mixtures containing equal concentrations of the three targeted species were applied in the multiplex PCR at final concentrations of 1, 0.5, 0.1, 0.05, 0.01, 0.005, 0.001 ng/μl to determine the sensitivity of the assay.

### Applicability for the detection of human and animal infection

A number of clinical lesion samples, as well as suspected lesions separated from animals, and feces harvested from purged owned dogs were used for further assessment of the assay. DNA extracted from all the isolates were randomly re-coded and then tested blindly.

## Results

### Primer design and selection

Having tested several primers and their combinations, three pairs of primers target on ATPase subunit 6 (*atp6*) gene and its flanking region of *E. granulosus* s. s., NADH dehydrogenase subunit 1 (*nd1*) gene of *E. multilocularis*, and large ribosomal RNA (*rrnL*) gene of *E. canadensis* were chosen for multiplex PCR amplification. The information of the primers used in the method was presented in Table 3.

### Detection of single and mixed infection

**Fig. 1:**
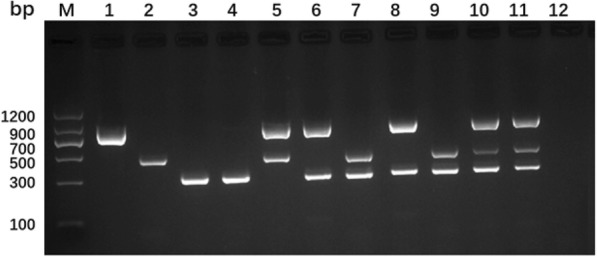
Multiplex PCR products from *E. granulosus* s. s., *E. multilocularis*, *E. canadensis* (G6/7 and G8/10) and their combinations. Lane M, Molecular weight markers (DNA marker II, Tiangen Biotech); lane 1, *E. granulosus* s. s.; lane 2, *E. multilocularis*; lane 3, *E. canadensis* (G6/7); lane 4, *E. canadensis* (G8/10); lane 5, *E. granulosus* s. s. and *E. multilocularis*; lane 6, *E. granulosus* s. s. and *E. canadensis* (G6/7); lane 7, *E. multilocularis* and *E. canadensis* (G6/7); lane 8, *E. granulosus* s. s. and *E. canadensis* (G8/10); lane 9, *E. multilocularis* and *E. canadensis* (G8/10); lane 10, *E. granulosus* s. s., *E. multilocularis* and *E. canadensis* (G6/7); lane 11, *E. granulosus* s. s., *E. multilocularis* and *E. canadensis* (G8/10); lane 12, no template control

The designed primer combination is able to discriminate among *E. granulosus* s. s., *E. multilocularis*, and *E. canadensis*, and to detect the random mixtures of their DNA simultaneously in one-tube reaction (Fig. 1). Each pair of primers generated specified products with expected size which presented clear and distinct bands on agarose gel. Using primer c/f and c/r, the multiplex PCR assay identified the genotype 6/7 and genotype 8/10 of *E. canadensis* resulting in bands with the same size.

### Specificity and sensitivity of the multiplex PCR

**Fig. 2:**
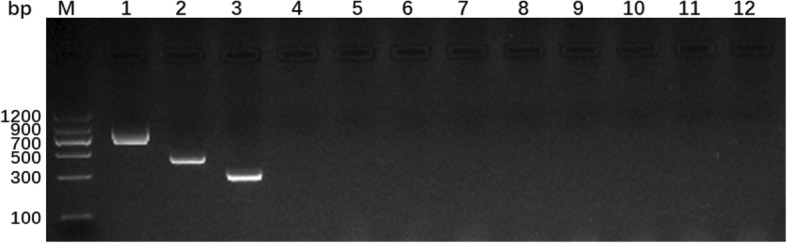
Multiplex PCR products from three targeted and eight genetically related but non-targeted parasites. Lane M, Molecular weight markers (DNA marker II, Tiangen Biotech); lane 1, *E. granulosus* s. s.; lane 2, *E. multilocularis*; lane 3, *E. canadensis* (G6/7); lane 4, *E. shiquicus*; lane 5, *T. saginata*; lane 6, *T. solium*; lane 7, *T. asiatica*; lane 8, *T. serialis*; lane 9, *T. taeniaeformis*; lane 10, *T. pisformis*; lane 11, *H. diminuta*; lane 12, no template control

**Fig. 3:**
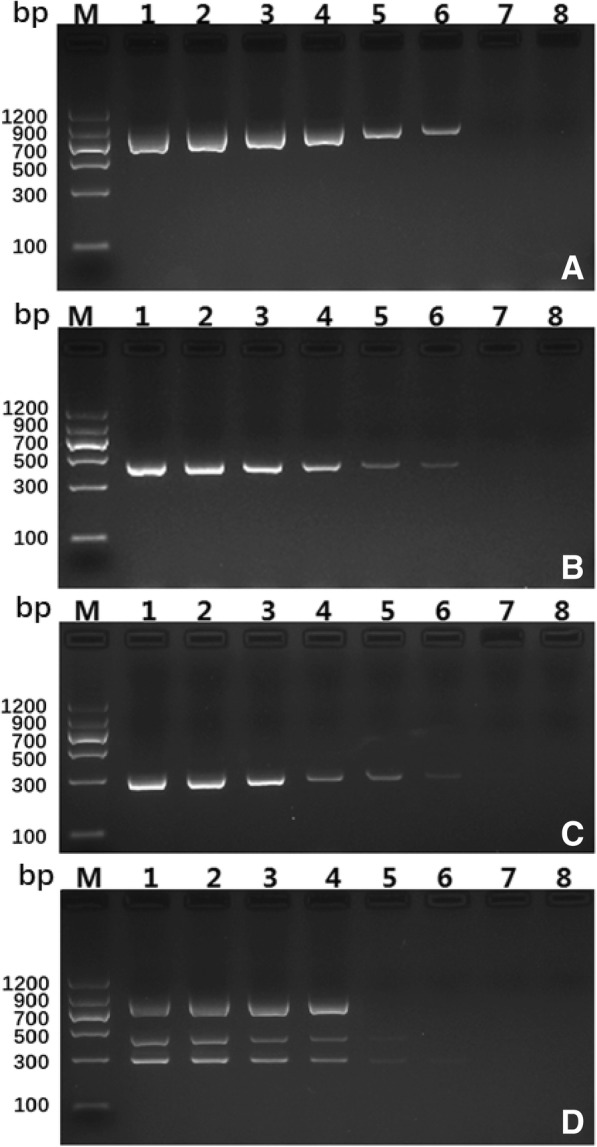
Multiplex PCR products from (**a**) *E. granulosus* s. s., (**b**) *E. multilocularis*, (**c**) *E. canadensis* (G6/7) and (**d**) their mixtures. Lane M, Molecular weight markers (DNA marker II, Tiangen Biotech); lane 1, DNA templates with final concentration of 1 ng/μl; lane 2, DNA templates with final concentration of 0.5 ng/μl; lane 3, DNA templates with final concentration of 0.1 ng/μl; lane 4, DNA templates with final concentration of 0.05 ng/μl; lane 5, DNA templates with final concentration of 0.01 ng/μl; lane 6, DNA templates with final concentration of 0.005 ng/μl; lane 7, DNA templates with final concentration of 0.001 ng/μl; lane 8, No template control

No cross-reaction was observed when DNA templates adopted from eight relevant but non-targeted parasites within the family *Taeniidae* were used (Fig. 2). The amplicons of *E. granulosus* s. s., *E. multilocularis*, and *E. canadensis* were detectable with amount as low as 5 pg/μl when DNA from each of the targeted species was tested individually (Fig. 3). When the assay was performed using the templates consisted of DNA mixtures of these three species at the same concentration, the detection limit was 50 pg/μl, 10 pg/μl and 5 pg/μl for *E. granulosus* s. s., *E. multilocularis*, and *E. canadensis* respectively (Fig. 3).

### Detection of human and animal infection samples

The developed multiplex PCR method was used to test samples obtained from patients and different infected animals. The detection results were shown in Table 4.

**Table 4:** Detection results of the samples used to validate the applicability of the multiplex PCR

Host	Species	Multiple PCR identification			
		*Echinococcus granulosus* s. s.	*E. multilocularis*	*E. canadensis*	Negative
Human (*n* = 36)	*E. granulosus* s. s.	22	0	0	0
	*E. multilocularis*	0	13	0	0
	*E. canadensis*(G6/7)	0	0	1	0
Yak (*n* = 14)	*E. granulosus* s. s.	14	0	0	0
Small mammal (*n* = 23)	*E. multilocularis*	0	7	0	0
	*E. shiquicus*	0	0	0	16
Dog (*n* = 41)	*E. granulosus* s. s.	1	0	0	0
	*E. multilocularis*	0	26	0	0
	*E. shiquicus*	0	0	0	1
	Negative	0	0	0	13

DNA isolated from parasite lesions taken from humans and domestic animals was all successfully amplified, and the multiplex PCR identification results agreed with the initial sequencing results.

Additionally, seven out of 23 samples derived from small mammals were detected as *E. multilocularis*, which are consistent with previous sequencing results. The other 16 samples pre-identified as *E. shiquicus* by sequencing presented negative result in the multiplex PCR.

The multiplex PCR results for the fecal samples were in agreement with the pre-sequencing records, except one, which showed negative signals in the assay was previously determined as *E. shiquicus*.

Furthermore, of all the tested stool samples, 16 were found to contain adult worms of *Echinococcus* by visual and microscopic examination. Among the 16 positive samples, 13 were detected as *E. multilocularis* by the multiplex PCR, and the remained three presented negative both in the current assay and previous molecular identification. Moreover, 14 microscopic-negative samples generated positive signals in the multiplex PCR (Table 5) .

**Table 5:** Comparison of the results of the fecal materials between visual / microscopic examination and molecular detections

Visual and microscopic examination	Pre-sequencing				Multiple PCR identification		
	*Echinococcus granulosus* s. s.	*E. multilocularis*	*E. shiquicus*	Negative	*E. granulosus* s. s.	*E. multilocularis*	Negative
Positive	0	13	0	3	0	13	3
Negative	1	13	1	10	1	13	11
Total	1	26	1	13	1	26	14

## Discussion

Echinococcosis is one of the biggest public health challenges in China. The species composition of the causative agents leading to the disease transmitted in this area is rather complex [6, 26–28]. At least four *Echinococcus* species are proven to be co-endemic, 3 of which are related to human echinococcosis. To achieve rapid diagnosis and targeted prevention of echinococcosis, it is necessary to develop an accurate and efficient method to identify the species for clinical and epidemiological use.

In the study, a fast and convenient multiplex PCR assay was established to identify and discriminate *E. granulosus* s. s., *E. multilocularis*, and *E. canadensis* in a single tube reaction. Closely related parasite species which commonly detected in the same animal hosts inhabit in the endemic areas of Sichuan were picked to test the cross-reactivity of the assay. No false positive signals were detected among them. However, further validation were suggested if the assay is applied to other endemic regions with the existence of any other related parasites that may result in non-specific amplifications in the multiplex PCR system.

Domestic animals are known to be the main intermediate hosts of *E. granulosus* s. s., whilst *E. multilocularis* and *E. shiquicus* are harbored by small mammal hosts. The results of the validation suggested that the developed method possesses an ideal capability of detecting the first two targeted species, and does not show any cross-reactivity with the third species testified by multiplex PCR-negative in the study. *E. shiquicus* is a newly discovered species that appears to be exclusive in China [12]. For now, no evidence was found to indicate it has any pathogenic potential to humans. Therefore, this species is not taken as the detection target in the study since it is not the primary concerns in the control program. However, of course, the method could be further improved to a quadruplex PCR utilized in ecological or biological investigation of *Echinococcus* species by adding a pair of *E. shiquicus*-specific primers.

The advantage of the multiplex PCR method becomes more obvious when it is used for the test of the causative pathogens in definitive hosts other than intermediate hosts, because miss detection of mixed species infections are more likely to happen in stools compared to tissues, even though this kind of samples count just for a small portion of the total. None of the fecal materials showed co-infection in the study owing to the lack of such samples, but the results of the spiked DNA samples have fully proved that the developed assay is capable of detecting double and triple mixed infections of the targeted species effectively.

However, the sensitivity of this assay is considered to be not good enough when it is applied to detect the infection in canine faces. Basing on the data that nuclear DNA is about 8 pg per egg [29], at least 15 eggs are need to generate a positive result using the developed method, even more for mixed infections. Therefore, to avoid false negative result from low infection, the enlargement of test volume is suggested to enrich the pathogens in feces. Previously reported multiplex PCR methods showed higher sensitivities, but they were targeted on *E. shiquicus, Taenia* spp*.* and *E. granulosus* s.l. [21, 22].

The evaluation results based on various samples obtained from human and different animal hosts indicated that the multiplex PCR developed in this study is applicable for clinical and epidemiological use, particularly for the confirmation and classification of human echinococcosis. Further optimization will be implemented.

In addition, similar as reported before [22], a weak correlation was observed between the results of purgation and the multiplex PCR. Fourteen fecal materials which had no *Echinococcus* adults examined by naked eyes and microscope showed positive bands in the multiplex PCR. The followed sequencing results exclude the possibility that the amplicons yielded from non-specific amplification. A previous study revealed that about 40% dogs with *Echinococcus* infections were not sensitive to arecoline purgation resulting in false negatives (absence of worms in purged excretions) [30]. The results in the study suggested that the developed assay could detect this kind of purge-negative cases which might result from low infection intensities. On the other hand, three samples contained *Echinococcus* worms showed false negative outcomes in the multiplex PCR. Since none of these samples gave positive signals at the initial molecular identification either, the inconsistence might be due to the inhomogeneous distribution of the pathogens in feces.

As increasingly being identified in recent years, genotype G6/7 is now believed to have a much broader geographical distribution, wider host range and more significant impacts on human health in China and worldwide [6, 31, 32]. Three cases of genotype G8/10 infection were documented in China at present, two of which found on the Qinghai-Tibetan Plateau indicated a high probability that the transmission of this genotype occurs in the area [27, 33, 34]. Since the distribution and prevalence of genotype G6/7 and G8/10 in China are still unclear, thus the identification of them should be taken into account within the routine diagnosis and surveillance system.

The taxonomic status of *E. canadensis* is highly controversial, which genotype G6/7 and G8/10 tend to be regarded as two different species now [35]. However, the proposal of revision has not reach a consesus yet. Since toxomony is not the primary research issue in the study, and the number of cases caused by the infection of the two genotypes especially G8/10 are much lower compared to *E.granulosus* s. s. and *E.multilocularis*. Therefore, the study is aimed at detecting the genotype G6/7 and G8/10 at the same time without distinguishment. DNA from the genotype G6/7 and G8/10 were both used to validate the multiplex PCR. The result showed that the two DNA samples were successfully amplified and generated PCR products with same expected size on gel. Both the genotype G6/7 and G8/10 can be identified in the developed assay just as designed. It should be noted that, however, the distribution and host selection of the two genotypes are known to be different [36], and human response to them may vary too. The discrimination between the genotype G6/7 and G8/10 is needed eventually. Besides, thus far samples of these genotypes obtained to verify this assay were limited, further examinations with more specimens are required.

## Conclusions

The new single-tube multiplex PCR method that allows rapid, accurate and simultaneous detection of *E. granulosus s. s.*, *E. multilocularis* and *E. canadensis* was developed and assessed in the study. The results suggested that the method has potential application in fast detection and large-scale screening, which makes it a promising candidate as the key technology for clinical diagnosis and environmental monitoring.

## Additional file

Additional file 1: Multilingual abstracts in the five official working languages of the United Nations. (PDF 271 kb)

## Data Availability

The data supporting the conclusions of this article are included within the article.

## Abbreviations

AE: Alveolar echinococcosis
BLAST: Basic local alignment search tool
CE: Cystic echinococcosis
DNA: Deoxyribonucleic acid
NCBI: National center for biotechnology information
PCR: Polymerase chain reaction
