# Supplementary material for: A multiplex PCR for differential detection of Echinococcus granulosus sensu stricto, Echinococcus multilocularis and Echinococcus canadensis in China
Source: Infect Dis Poverty. 2019 Jul 30;8:68. doi: 10.1186/s40249-019-0580-2 (PMC6668063; doi:10.1186/s40249-019-0580-2)
Supplement: Supplementary file 1 — Multilingual abstracts in the five official working languages of the United Nations. (PDF 271 kb) [file 40249_2019_580_MOESM1_ESM.pdf]

Translation of the abstract into the five official working languages of the United Nations

جهاز pcr وهو جهاز الكشف عن الأنواع والأنماط الجينية لمرض المشوكات الرئوية والذي يسببه طفيل المشوكة الحبيبية، وهو احد أكبر الأوبئة في مناطق شمال غرب الصين.

جينغ يي شانغ، غوانغ جيا تشانغ، شا لياو، يان هوانغ، وين جي يو، وي هي، غوانغ يو يانغ، تياو يينغ لي، شينغ وانغ تشن، بو تشونغ، تشيان وانغ، تشى وانغ روي لي، هاو وانغ

نبذة مختصرة

الخلفية: ما زال داء المشوكة الرئوية أحد أكبر الأمراض المعدية المنتشرة في شمال غرب مناطق مرتفعات الصين. داء المشوكة الكيسية الهيدريات، داء المشوكة السنخية وداء المشوكة الكيسية المتعددة وهي الأنواع الثلاثة المرتبطة بصحة الانسان وتنتقل إليه في تلك المناطق. ولتحقيق العلاج المستهدف والسيطرة على المرض يكون التحديد الدقيق والتمييز للأنواع بالغ الأهمية. ومع ذلك، لا تقدم طرق التشخيص المتوفرة حاليًا نتائج مثالية سواء في الدقة أو الكفاءة.

الطريقة: في الدراسة، تم تصميم مجموعة من الاشغال لتهدف إلى ثلاثة أنواع مسببة للأمراض البشرية في الصين. تم تطوير اختبار PCR متعدد الخطوات في خطوة واحدة وتقييم لخصوصيته وحساسيته. تم اختبار ما مجموعه 73 آفة طفيلية و 41 مادة برازية تم الحصول عليها من بشر وحيوانات مختلفة تم جمعها في العيادة والميدان لتقييم مدى قابلية تطبيق هذه الطريقة.

النتائج: اكتشف PCR على نحو فعّال الحمض النووي الفردي من الأنواع المستهدفة وامتزاجاتها العشوائية التي تولد مع حجم متوقع يمكن تمييزه من المنتجات. كان الحد الأقصى لاكتشاف الفحص لكل نوع من الأنواع الثلاثة هو 5 بيكوغرام / ميكرولتر عند اختباره بشكل منفصل. عندما تم استخدام خليط الحمض النووي من الأنواع المستهدفة التي تحتوي على نفس التركيز كقوالب، فإن أقل كمية من الحمض النووي التي يمكن اكتشافها كانت 50 بيكوغرام / ميكرولتر، 10 بيكوغرام / ميكرولتر و 5 بيكوغرام / ميكروليتر ل E. granµE. granµLosus ss و E. mµLtilocµLaris و E. canadensis تباعاً. لم يُلاحظ أي تفاعل متبادل عندما تم استخدام الحمض النووي من ثمانية أنواع قريبة وراثياً كقوالب السيطرة. كانت تعريفات PCR المتعددة لجميع العيّنات متماشية مع نتائج التسلسل الأصلي باستثناء المصابين بفيروس E. shiquicus، والتي أظهرت إشارات سلبية في الاختبار المتطور. من بين جميع المواد التي تم اختبارها في البراز، تم العثور على 16 منها في وقت سابق لداء المشوّكات عن طريق الفحص البصري والمجهر. من بين هذه العينات الـ 16، تم تأكيد 13 حالة بواسطة PCR، وتم اختبار النتائج الثلاثة السلبية الأخرى. بالإضافة إلى ذلك، حدد PCR multiplex 14 براز إيجابي آخر من عينات البراز الـ25 المتبقية التي تفتقر إلى الديدان.

الاستنتاجات: يُظهر PCR المتطور مزايا في التشخيص السريع والتحقيق الوبائي واسع النطاق، والذي أثبت أنه أداة واعدة تستخدم في العيادة ونظام المراقبة.

Translated from English version into Arabic by Hanan Awd, Revised by Sophie Chammas, through

TRANSLATORS
WITHOUT BORDERS

一种用于中国地区狭义细粒棘球绦虫、多房棘球绦虫和加拿大棘球绦虫的多重 PCR 检测方法

尚婧晔，张光葭，廖沙，黄燕，喻文杰，何伟，杨光友，李调英，陈兴旺，钟波，王谦，王奇，李泐芮，汪浩

摘要

引言：棘球绦虫引起的棘球蚴病是中国西北高原最主要的传染病之一。狭义细粒棘球绦虫、多房棘球绦虫和加拿大棘球绦虫是已知的在该地区传播的唯一三种与人类健康有关的棘球绦虫种。为实现棘球蚴病的精准防治，虫种的准确鉴别和区分显得尤为重要的。然而，现有的诊断方法不论是准确性或其效率性均不甚理想。

方法：本研究针对中国地区流行的三种对人类具有致病性的棘球绦虫设计引物，建立一步多重 PCR 检测方法，并对其特异性和灵敏度进行评估。通过对从临床和现场收集的、来源于人和各种动物的共计 73 份寄生虫病灶和 41 份粪便样本进行检测，用以评估该方法的适用性。
结果：多重 PCR 可有效地检测出来自目标虫种的单独 DNA 及其随机混合物，并扩增出片段长度为预期大小并且可区分的产物。对三个虫种分别检测时，其检测限均为 5 pg /µl。使用含有相同浓度的三种目标虫种的 DNA 混合物作为模板时，狭义细粒棘球绦虫、多房棘球绦虫和加拿大棘球绦虫的 DNA 最低检出量分别为 50 pg /µl、10 pg /µl 和 5 pg /µl。以 8 种亲缘关系较近的虫种 DNA 作对照模板时，未观察到交叉反应性。除了石渠棘球绦虫样本多重 PCR 检测结果为阴性外，其它所有样品的多重 PCR 鉴定结果与原始测序结果相符合。所有的犬粪样本此前已通过目视和显微镜检查发现了 16 份棘球绦虫阳性。这 16 份样品中，13 个通过多重 PCR 得到了确认，另外 3 个样本多重 PCR 检测结果为阴性。此外，余下的 25 份此前未发现有虫体存在的粪便样品中，有 14 份多重 PCR 检测结果为阳性。
结论：建立的多重 PCR 在快速诊断和大规模流行病学调查方面具有优势，是一种可用于临床和监测体系中的具有前景的检测工具。

Translated from English version into Chinese by Jing-Ye Shang

Une PCR multiplex pour la détection différentielle d' *Echinococcus granulosus* sensu stricto, d' *E. Multilocularis* et d' *E. Canadensis* en Chine

Jing-Ye Shang, Guang-Jia Zhang, Sha Liao, Yan Huang, Wen-Jie Yu, Wei He, Guang-You Yang, Tiao-Ying Li, Xing-Wang Chen, Bo Zhong, Qian Wang, Qi Wang，Rui-Rui Li, Hao Wang

Résumé
Contexte: L'échinococcose causée par *l'échinocoque* est l'une des principales maladies infectieuses dans les hauts plateaux du nord-ouest de la Chine. *E. granulosus* sensu stricto, *E. multilocularis*, et *E. canadensis* sont connues pour être les trois seules espèces en relation avec la santé humaine qui transmettent dans ces zones. Pour parvenir à un traitement et à un contrôle ciblés de l'échinococcose, il est important d'identifier et de distinguer précisément l'espèce. Cependant, à l'heure actuelle, les approches diagnostiques disponibles ne présentent pas de résultats idéaux, ni en termes de précision, ni en termes d'efficacité.
Méthodes: Dans l'étude, un ensemble d'amorces a été conçu pour cibler les trois espèces pathogènes pour l'homme *Echinococcus*en Chine. Le test PCR multiplex à une étape a été développé et évalué pour sa spécificité et sa sensibilité. Au total, 73 lésions parasitaires et 41 matières fécales provenant d'humains et de divers animaux prélevés en clinique et sur le terrain ont été testées pour évaluer la pertinence de cette méthode.
Résultats: La PCR multiplex a détecté efficacement l'ADN individuel de l'espèce ciblée et ses mélanges aléatoires générant une taille attendue distincte des produits. La limite de détection du test pour chacune des trois espèces était de 5 pg/µL lorsqu'elles ont été testées séparément. Lorsque des mélanges d'ADN des espèces ciblées contenant la même concentration ont été utilisés comme modèles, la plus faible quantité d'ADN pouvant être détectée était de 50 pg/µL, 10 pg/µL et 5 pg/µL pour *E. granµLosus* s. s., *E. mµLtilocµLaris*, et *E. canadensis* respectivement. Aucune réactivité croisée n'a été observée lorsque l'ADN de huit espèces génétiquement proches a été utilisé comme modèle témoin. Les identifications PCR multiplex de tous les échantillons étaient en ligne avec les résultats de séquençage originaux à l'exception de ceux infectés par *E. shiquicus*, qui ont présenté des signaux négatifs dans le test développé. De tous les matériaux de selles testés, 16 étaient auparavant positifs pour *Echinococcus* par examen visuel et microscopique. Parmi ces 16 échantillons, 13 ont été confirmés par PCR multiplex et les trois autres se sont révélés négatifs. De plus, la PCR multiplex a identifié 14 autres selles positives dans les

25 échantillons de selles restants, ce qui signifie l'absence de vers.
Conclusions: La PCR multiplex développée présente des avantages dans le diagnostic rapide et l'investigation épidémiologique à grande échelle, qui s'est avérée être un outil prometteur utilisé en clinique et dans les systèmes de surveillance.

Translated from English version into French by Codina Charles, Revised by Imane Bouamoud, through

Мультиплексная ПЦР для дифференциального определения *Echinococcus granulosus* в узком смысле, *E. multilocularis* и *E. canadensis* в Китае

Цзин-Е Шан, Гуан-Цзя Чжан, Ша Ляо, Янь Хуан, Вень-Цзе Юй, Вэй Хэ, Гуан-Ю Ян, Тяо-Ин Ли, Син-Ван Чэнь, Бо Чжун, Цянь Ван, Ци Ван, Жуй-Жуй Ли, Хао Ван

Аннотация

Предпосылки: Эхинококкоз, вызванный *эхинококком,* является одним из наиболее распространенных инфекционных заболеваний на северо-западе Китая. Известно, что *E. granulosus* в узком смысле, *E. multilocularis* и *E. canadensis*являются единственными тремя видами, связанными с заболеваемостью людей в этих районах. Для целенаправленного лечения и контроля эхинококкоза важна точная идентификация и распознавание видов. Однако в настоящее время доступные диагностические подходы не дают идеальных результатов ни по точности, ни по эффективности.
Методы: В исследовании был разработан набор праймеров, предназначенных для трех патогенных для человека видов *эхинококков* в Китае. Был разработан и оценен одностадийный мультиплексный ПЦР-анализ на специфичность и чувствительность. В общей сложности 73 паразитарных очага и 41 фекальный материал, полученные от людей и различных животных, собранные в клинике и на местах, были протестированы для оценки применимости этого метода.
Результаты: Мультиплексная ПЦР эффективно обнаружила отдельные ДНК из целевых видов и их случайные смеси, различаемые по ожидаемым размерам продуктов. Порог обнаружения анализа для каждого из трех видов составлял 5 пг/мкл, когда они тестировались отдельно. Когда в качестве матриц использовали смеси ДНК целевых видов, содержащие одинаковую концентрацию, наименьшее количество ДНК, которое можно обнаружить, составляло 50 пг/мкл, 10 пг/мкл и 5 пг/мкл для *E. granulosus* s. s., *E. multilocularis*и *E. canadensis* соответственно. Никакой перекрестной реактивности не наблюдалось, когда ДНК из восьми генетически близких видов использовалась в качестве контрольных матриц. Мультиплексная ПЦР-идентификация всех образцов соответствовала исходным результатам секвенирования, за исключением тех, которые были инфицированы *E. shiquicus*, которые показали отрицательные сигналы в разработанном анализе. Из всех исследованных материалов стула 16 были ранее признаны положительными на *эхинококк* при визуальном и микроскопическом исследовании. Из этих 16 образцов 13 были подтверждены мультиплексной ПЦР, а три других были отрицательными. Кроме того, мультиплексная ПЦР идентифицировала еще 14 положительных фекалий из оставшихся 25 образцов кала, в которых отсутствовали глисты.
Выводы: Разработанная мультиплексная ПЦР демонстрирует преимущества в быстрой диагностике и широкомасштабных эпидемиологических исследованиях, что оказалось перспективным инструментом для использования в клиниках и системах эпиднадзора.

Translated from English version into Russian by Anna Kukharchuk, Revised by Alexander Somin, through

TRANSLATORS
WITHOUT BORDERS

La PCR múltiple para la detección diferencial de *Echinococcus granulosus* sensu stricto, *E. multilocularis* y *E. canadensis* en China

Jing-Ye Shang, Guang-Jia Zhang, Sha Liao, Yan Huang, Wen-Jie Yu, Wei He, Guang-You Yang, Tiao-Ying Li, Xing-Wang Chen, Bo Zhong, Qian Wang, Qi Wang, Rui-Rui Li, Hao Wang

Resumen

Antecedentes: la equinococosis causada por el *Echinococcus* es una de las principales enfermedades infecciosas en las zonas montañosas del noroeste de China. Se sabe que *E. granulosus* sensu stricto, *E. multilocularis* y *E. canadensis* son las tres únicas especies relacionadas con la salud humana que se transmiten en las áreas mencionadas. La identificación y discriminación precisas de las especies son importantes para lograr el tratamiento específico y el control de la equinococosis. Sin embargo, en la actualidad, los enfoques de diagnóstico disponibles no proporcionan resultados ideales, ni en precisión ni en eficiencia.

Métodos: en el estudio se diseñó un conjunto de cebadores específicos para las tres especies de *Echinococcus* patógenas para el hombre en China. El ensayo de PCR múltiple de un solo paso se desarrolló y evaluó para determinar su especificidad y sensibilidad. Se realizaron pruebas en un total de 73 lesiones parasitarias y 41 materias fecales procedentes de seres humanos y de diversos animales para evaluar la aplicabilidad del método. Las muestras fueron recolectadas tanto en la clínica como en el campo.

Resultados: la PCR múltiple detectó de manera eficaz el ADN individual de las especies objetivo y sus mezclas aleatorias, generando el tamaño distinguible esperado de los productos. El límite de detección del ensayo para cada una de las tres especies, cuando se probaron por separado, fue de 5 pg/μL. Cuando se utilizaron como modelos mezclas de ADN de las especies objetivo que contenían las mismas concentraciones de cada una de ellas, las menores cantidades de ADN que se pudieron detectar fueron de 50 pg/μL, 10 pg/μL y 5 pg/μL para *E. granulosus* s. s., *E. multilocularis* y *E. canadensis*, respectivamente. No se observó reactividad cruzada cuando de empleó como modelo de control el ADN de ocho especies genéticamente parecidas. Las identificaciones de la PCR múltiple de todas las muestras concordaron con los resultados de la secuencia original, salvo las infectadas por *E. shiquicus*, que dieron resultados negativos en el ensayo realizado. De todas las materias fecales sometidas a ensayo, 16 dieron positivo para *Echinococcus* en un examen visual y microscópico realizado con anterioridad. De esas 16 muestras, 13 fueron confirmadas mediante PCR múltiple, y las otras 3 dieron negativo. Además, la PCR múltiple detectó otras 14 heces positivas de entre las 25 muestras fecales restantes que no presentaban lombrices.

Conclusiones: la PCR múltiple que se llevó a cabo muestra ventajas en el diagnóstico rápido y en la investigación epidemiológica a gran escala, y ha demostrado ser una herramienta prometedora para que sea empleada en el sistema clínico y de vigilancia.

Translated from English version into Spanish by Celia Martinez, Revised by Mayra León, through

TRANSLATORS
WITHOUT BORDERS
